# Supplementary material for: Sex and APOE Genotype Alter the Basal and Induced Inflammatory States of Primary Microglia from APOE Targeted Replacement Mice
Source: Int J Mol Sci. 2022 Aug 29;23(17):9829. doi: 10.3390/ijms23179829 (PMC9456163; doi:10.3390/ijms23179829)
Supplement: Supplementary file 1 [file ijms-23-09829-s001.zip › ijms-1850673-supplementary.pdf]

Table S1: Primer sequences

Il1b	Forward	AGTTGACGGACCCCAAAAGAT
	Reverse	GGACAGCCCAGGTCAAAGG
Il6	Forward	TCCATCCAGTTGCCTTCTTG
	Reverse	ATTGCCATTGCACAACTCTTTT
Tnfa	Forward	AGGGATGAGAAGTTCCCAAATG
	Reverse	TGTGAGGGTCTGGGCCATA
Il10	Forward	AGGCAGCCTTGCAGAAAAGA
	Reverse	AGTAAGAGCAGGCAGCATAGCA
Nos2	Forward	TCACGCTTGGGTCTTGTT
	Reverse	CAGGTCACTTTGGTAGGATTT
Ifng	Forward	ATGAAAATCCTGCAGAGCCA
	Reverse	GTGGGTTGTTGACCTCAAACT
Mcp1	Forward	TTGAATGTGAAGTTGACCCGTAA
	Reverse	GCTTGAGGTTGTGGAAAAG
Arg1	Forward	GGACCTGGCCTTTGTTGATG
	Reverse	AGACCGTGGGTTCTTCACAATT
Igf1	Forward	CGCCTCATTATCCCTGCCCACCA
	Reverse	GCCATAGCCTGTGGGCTTGTTGAA
Ym1	Forward	TCTGGTGAAGGAAATGCGTAAA
	Reverse	GCAGCCTTGGAATGTCTTTCTC
Mrc1	Forward	CCCAAGGGCTCTTCTAAAGCA
	Reverse	CGCCGGCACCTATCACA
Tgfb	Forward	GAGCCCGAAGCGGACTACT
	Reverse	TGCGGTCCACCATTAGCA
Fizz1	Forward	CAGCTGATGGTCCCAGTGAA
	Reverse	TTCCTTGACCTTATTCTCCACGAT
Rpl13a	Forward	CTGTGAAGGCATCAACATTTCTG
	Reverse	GACCACCATCCGCTTTTTCTT
Actin	Forward	TCATTGCTCCTCCTGAGCGCAA

	Reverse	GCAGCTCAGTAACAGTCCGCCTAG
Sry	Forward	TTGTCTAGAGAGCATGGAGGGCCATGTCAA
	Reverse	CCACTCCTCTGTGACACTTTAGCCCTCCGA
